# Investigation of Anti-Cancer Properties of Nano-Encapsulated Ciprofloxacin Using 3D Cancer Cell Spheroids as Tumour Models

**DOI:** 10.3390/ijms26125530

**Published:** 2025-06-10

**Authors:** Yasmin Kabalan, Karolina Matulewicz, Bartosz Tylkowski, Marta Woźniak-Budych, Katarzyna Staszak, Xavier Montané, Anna Bajek

**Affiliations:** 1 Department of Chemical Engineering, Universitat Rovira i Virgili, Av. Països Catalans 26, 43007 Tarragona, Spain; yasmin.kabalan@urv.cat (Y.K.); bartosz.tylkowski@eurecat.org (B.T.); 2 Faculty of Medicine, Bydgoszcz University of Science and Technology, Aleje Prof. S. Kaliskiego 7, 85-796 Bydgoszcz, Poland; karolina.matulewicz@pbs.edu.pl; 3 Department of Clinical Neuropsychology, Nicolaus Copernicus University in Toruń, The Ludwik Rydygier Collegium Medicum in Bydgoszcz, ul. Skłodowskiej Curie 9, 85-094 Bydgoszcz, Poland; 4 Eurecat, Centre Tecnològic de Catalunya, Unitat de Tecnologia Química, Marcel·lí Domingo 2, 43007 Tarrgona, Spain; 5 NanoBioMedical Centre, Adam Mickiewicz University, Wszechnicy Piastowskiej 3, 61-614 Poznan, Poland; marta.budych@amu.edu.pl; 6 Institute of Chemical Technology and Engineering, Faculty of Chemical Technology, Poznan University of Technology, ul. Berdychowo 4, 60-965 Poznan, Poland; katarzyna.staszak@put.poznan.pl; 7 Department of Analytical Chemistry and Organic Chemistry, Universitat Rovira i Virgili, C/Marcel·lí Domingo 1, 43007 Tarragona, Spain; xavier.montane@urv.cat; 8 Department of Oncology, The Ludwik Rydygier Collegium Medicum in Bydgoszcz, Nicolaus Copernicus University in Torun, ul. Łukasiewicza 1, 85-821 Bydgoszcz, Poland

**Keywords:** 3D tumour spheroids, ciprofloxacin, chitosan

## Abstract

Bladder cancer remains a significant global health challenge, necessitating innovative therapeutic strategies to enhance treatment efficacy. This study investigates the potential of chitosan nanoparticles as a drug delivery system, using ciprofloxacin as a model compound and utilizing a 3D spheroid model of bladder cancer that better reflects in vivo tumour conditions. The encapsulation efficiency of ciprofloxacin was optimized on the appropriate mass ratio of chitosan to cross-linking tripolyphosphate (TPP) polyanion. The resulting spherical chitosan nanocapsules loaded with ciprofloxacin demonstrated improved stability and controlled drug release, addressing the limitations of non-encapsulated ciprofloxacin. In 3D T24 bladder cancer spheroids, encapsulated ciprofloxacin exhibited enhanced cytotoxicity compared to free-drug formulations, with significant effects observed at ciprofloxacin concentrations of 500 and 1000 μM after 48 and 72 h of exposure. The 3D spheroid model, which better mimics the tumour microenvironment than 2D cultures, enabled a more accurate drug efficacy evaluation. The results demonstrate that chitosan nanocapsules can improve the delivery and cytotoxic profile of ciprofloxacin in vitro, indicating their potential for further development as carriers in localized bladder cancer treatment.

## 1. Introduction

Cancer remains one of the most significant global health challenges, with millions of lives lost annually to this disease [1]. Among the various forms of cancer, bladder and prostate cancer are of particular concern, accounting for approximately 10% of all cancers worldwide [2]. It is anticipated that cancer cases are projected to increase by more than 50% by 2040 due to an ageing population and environmental factors, such as ethnicity, family history, and obesity [2,3]. For example, in Western countries, prostate cancer predominantly affects men aged between 45 and 60 years old [4]. Interestingly, men with bladder cancer are at increased risk of developing prostate cancer due to shared embryological origin and molecular similarities [5,6]. To gain insight into the mechanisms of cancer progression, it is necessary to employ innovative research tools capable of replicating the intricate tumour microenvironment. Traditional 2D cell cultures are unable to accurately replicate the spatial and biochemical intricacies of in vivo tumours [7]. In contrast, 3D tumour spheroid models provide a more realistic framework, resembling solid tumours in terms of their architectural composition, nutrient gradients, and oxygen distribution [8]. These models are fabricated using techniques such as hydrogel scaffolds, microfluidic technology, and 3D printing, and have emerged as essential tools for testing therapeutic strategies [9].

Ciprofloxacin (CIP), a broad-spectrum fluoroquinolone antibiotic, has demonstrated cytotoxic effects in in vitro cancer models, including bladder and prostate cancers [10]. Its ability to accumulate in the urine and urogenital tissues supports its consideration for local administration in urological conditions [11]. Its mechanism of cytotoxicity includes topoisomerase II inhibition, which leads to a disruption of DNA replication in rapidly dividing cancer cells. The selection of ciprofloxacin was based on its known activity against certain cancer cell lines and its accumulation in urogenital tissues, making it suitable for this proof-of-concept study.

**Figure 1:**
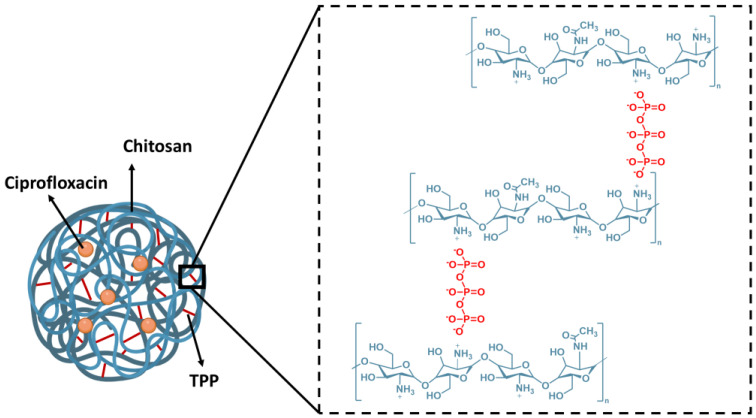
Chemical structure of cross-linking of chitosan chains by tripolyphosphate cations.

Nanotechnology offers promising solutions for overcoming challenges in cancer treatment. By leveraging biopolymer-based nanovehicles, it is possible to enhance drug stability, ensure controlled release, and reduce toxicity. Moreover, these systems offer additional advantages, including biodegradability, biocompatibility, and the ability to target specific tissues or cells. The choice of nanovehicle depends on different parameters, including the drug’s chemical structure, solubility, electrokinetic potential, and pharmacokinetics. Biopolymers have attracted great interest in the research community as nanovehicles due to their particular geometrical dimensions, high specific surface area, mechanical and barrier properties, lack of toxicity, biocompatibility, and biodegradability. Novel drug delivery systems based on biopolymers include chitosan, dextrin, polysaccharides, poly(glycolic acid), and hyaluronic acid [12]. As drug vehicles, biopolymers can protect drugs from degradation and ensure their prolonged release in physiological fluids. In these systems, the drug can be released from the biopolymer structure by diffusion, degradation, or swelling. Chitosan is a natural biopolymer that is biocompatible, non-toxic, and can form hydrogels [13]. It is derived from the deacetylation of chitin and consists of deacetylated D-glucosamine and *N*-acetyl-D-glucosamine linked by glycosidic bonds [14]. Under mild acidic conditions the deacetylated units of chitosan can serve as a source of cationic sites due to amino group protonation [15]. Several methods can be applied to formulate chitosan delivery vehicles, such as electrostatic self-assembly, lyophilization, complex coacervation, spray drying, and ionic gelation [16]. The ionic gelation is one of the simplest, fastest, and cheapest approaches to produce polymeric nano- or microparticles based on an electrostatic interaction between the opposite charges of the polymer- and polyanion-type compounds [17]. In this sense, it is expected that the cross-linking of chitosan chains by the tripolyphosphate (V) (TPP) polyanion could lead to the formation of chitosan-based nanoparticles. In the resulting structures, the protonated amine moieties of chitosan interact with negatively charged ions of TPP, creating stable ionic cross-linked networks, as shown in Figure 1 [18]. Chitosan has demonstrated significant potential as a carrier for bioactive compounds. In our previous studies, we utilized the spray-drying technique to encapsulate anti-proliferative polyphenols from *Cistus L.* [19] and folic acid [20]. Chitosan capsules enhance the therapeutic effect of drugs by improving bioavailability, controlling release, and reducing toxicity. Chitosan’s mucoadhesive properties support prolonged drug release, while its inherent antimicrobial properties and ability to enhance cellular uptake may provide synergistic effects with ciprofloxacin. Other researchers have developed ciprofloxacin delivery systems based on chitosan modifications, including grafted chitosan-coated zinc oxide nanoparticles [21] and methacrylated chitosan nanoparticles [22]. 

In this study, we present the encapsulation of ciprofloxacin in chitosan–TPP nanoparticles using the ionic gelation process (Figure 1) to optimize drug delivery. The chitosan composition and the ratio of chitosan to TPP were investigated to optimize the preparation of the nanoparticles and the encapsulation efficiency. We focus on the chitosan–TPP ratio to understand better the molecular interactions between cationic species of chitosan (protonated amino groups $-{NH}_{3}^{+}$) and triphosphate polyanions [P_3_O_10_]^5−^. This aspect has not been described in depth. We evaluate the anti-cancer potential of encapsulated ciprofloxacin in chitosan–TPP nanoparticles using an advanced 3D bladder cancer spheroids model fabricated via the magnetic 3D printing method. This innovative strategy bridges the gap between traditional in vitro models and the complex dynamics of tumour microenvironments, advancing the development of more effective cancer therapies. Research on ciprofloxacin in an antitumour context, particularly in 3D models, is still limited. Most of the previous work focuses on its activity in simpler in vitro models that do not reflect the complexity of the tumour environment. The study presented addresses this gap by using 3D spheroids that better simulate the tumour microenvironment, including oxygen gradients, nutrients, and cellular interactions. Furthermore, demonstrating the efficacy of encapsulated ciprofloxacin in this model indicates its potential in more complex biological systems.

## 2. Results and Discussion

Ciprofloxacin demonstrates potential anti-cancer activity in certain experimental contexts. However, its clinical application as an anti-cancer agent in conventional formulations faces significant limitations due to known adverse effects, including tendon damage and neurological complications. While these challenges make ciprofloxacin unlikely to replace established cancer therapies in its conventional form, targeted delivery systems may offer a solution by localizing its effects to tumour tissues while minimizing systemic exposure. Our study explores whether chitosan-based encapsulation could potentially modify ciprofloxacin’s delivery profile to overcome these limitations. The encapsulation process aims to achieve controlled release characteristics, potentially enhancing stability while reducing systemic adverse effects. Among various polymers, chitosan has emerged as a promising biopolymer for drug-delivery nanocarriers [23,24], offering advantages through its biocompatibility, biodegradability, and inherent antimicrobial properties [25]. Future studies would need to comprehensively evaluate both the anti-cancer efficacy and safety profile, including the potential impact on the gut microbiota resulting from antibiotic activity.

Usually, chitosan is obtained from chitin during a chemical process that involves applying a strong alkali, typically sodium hydroxide (NaOH), at high temperatures. This process removes the acetyl groups from the chitin molecule, converting it into chitosan. The extent to which acetyl groups are removed is measured as the degree of deacetylation (D.D.), and its value can vary depending on the production parameters used in its manufacture. The D.D. significantly influences chitosan’s physicochemical properties, including its solubility, charge density, and ability to form nanoparticles. Therefore, determining the concentration of ${-NH}_{3}^{+}$ groups in chitosan molecules is crucial for designing a drug nanoencapsulation process. The ratio between chitosan and TPP refers to the balance between the protonated amino groups (${-NH}_{3}^{+}$ of chitosan) and the polyanions of TPP (R-O^−^), which is critical for optimal nanoparticle formation and stability.

**Figure 2:**
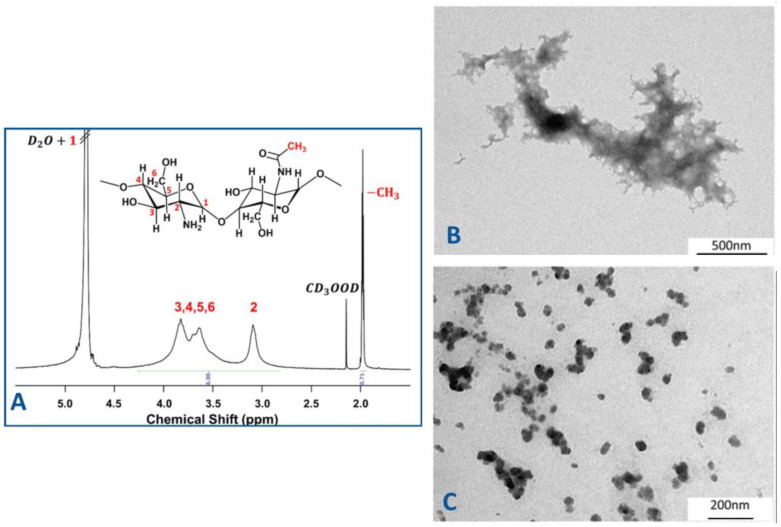
Characterization of chitosan and morphological analysis of chitosan nanoparticles formed at different [${-NH}_{3}^{+}$/[O^−^] ratios. (**A**) ^1^H NMR spectrum of chitosan in 2 wt.% CD_3_OOD/D_2_O; (**B**) TEM images of the structures obtained in the case of sample S1; (**C**) and sample S5. Sample compositions are detailed in Table 1.

In our studies, the chemical structure of chitosan was characterized by ^1^H NMR spectroscopy (Figure 2A), which confirmed the degree of deacetylation and provided insight into the distribution of functional ${-NH}_{3}^{+}$ groups. This characterization is essential for understanding the polymer’s behaviour in nanoparticle formation and its interaction with the encapsulated drug.

The ^1^H NMR spectrum is characterized by broad signals associated with the macromolecular structure of chitosan. The signal of protons indexed as H1 appears to overlap with the signal of D_2_O. The next signal centred between 3.30 and 4.10 ppm is attributed to the protons indexed as H3-H6 of the glucosamine moieties of D-glucosamine and N-acetyl-D-glucosamine, respectively. When we move to lower frequencies, the following broad signal centred at 3.2 ppm can be attributed to the protons indexed as H2, which are only present in the repetitive units of D-glucosamine. In the up-field region of the spectrum, the peaks at 2.15 and 1.99 ppm can be assigned to the methyl groups of deuterated acetic acid and the methyl groups of the acetylated glucosamine moieties of chitosan, respectively [26,27,28,29].

D.D. of the used chitosan was calculated according to the following Equation (1), which is based on the procedure reported by Hirai and coworkers [30]:

(1) $$D.D.\left(\%\right)=\left\{1-\left(\frac{1}{3}I_{\left(CH3\right)}/\frac{1}{6}I_{\left(H2-H6\right)}\right)\right\}\cdot 100$$

where I (CH_3_) is the integration value of the −CH_3_ groups of the *N*-acetylated moieties and the integration value of protons indexed as H2–H6.

The value of D.D. was found to be 76%. The concentration of −${NH}_{3}^{+}$ groups was calculated considering the D.D. value and the average molecular weight of applied chitosan. As shown in Figure 1, the nanocapsule structure is formed due to interactions between cations from chitosan (positively charged amino groups) and anions from TPP (negatively charged phosphate groups). This balance is critical for achieving optimal encapsulation efficiency and producing nanocapsules with uniform morphology and high drug-loading capacity.

Following the literature, the optimal pH for the drug encapsulation process using TPP is 4.7 because at this pH the polyanion is expected to be in anionic form, i.e., [H_2_P_3_O_10_]^3−^ [31], while ciprofloxacin is mainly in cationic from [32,33]. Moreover, at pH 4.7 the protonation degree of chitosan is 1 (which means that all amino groups are protonated because the pKa of chitosan is 6.2) [34,35].

Considering the variety of chitosan–TPP ratios (considering functional groups) and/or chitosan–TPP mass ratios used in the preparation of nanocapsules in the existing literature, in this work we investigated the impact of distinct stoichiometric ratios between cationic and anionic sites on the ciprofloxacin encapsulation efficiency. Five distinct formulations (S1–S5) were prepared, varying the ratios of chitosan to TPP. These formulations were then analyzed to evaluate encapsulation efficiency, as summarized in Table 1. It is important to underline that the calculated ratios provide initial insights into the interactions between $-{NH}_{3}^{+}$ and -O^−^ sites and that experimental factors, such as molecular conformation, steric hindrance and the shielding effects, can significantly impact the accessibility and reactivity of these functional sites [34].

The results presented in Table 1 indicate that the [${-NH}_{3}^{+}$]/[O^−^] ratio affects the drug encapsulation efficiency. At a lower value of [$-{NH}_{3}^{+}$]/[O^−^] ratio the encapsulation efficiency of ciprofloxacin is higher, moving from 9 to 22% of encapsulated ciprofloxacin (Table 1). The sample that does not follow this tendency is sample S3. The ciprofloxacin in this sample has not been encapsulated despite the increase in the TPP concentration, as suggested in the literature [36]. The low encapsulation efficiency (EE) of ciprofloxacin can be attributed to several physicochemical properties and process-related factors. Ciprofloxacin, although classified as hydrophobic under neutral conditions, exhibits increased solubility at acidic or basic pH due to its amphoteric nature. This property can hinder effective encapsulation, particularly in methods relying on hydrophobic interactions. Under acidic conditions, ciprofloxacin may dissolve in the aqueous phase, reducing its entrapment within hydrophobic core systems, such as nanoparticles or chitosan carriers. For example, Soliman et al. investigated ciprofloxacin hydrochloride (CHCl) encapsulation in chitosan nanoparticles via ionic gelation. Their results showed encapsulation efficiencies ranging from 23% to 45%, influenced by factors such as chitosan and tripolyphosphate concentrations and the ultrasonication process used during formulation. These findings align with reports of low drug retention, which is a common challenge in such nanoparticle systems due to the simplicity and variability of the ionic gelation method [37]. This study emphasizes the importance of optimizing the chitosan–TPP ratio and highlights the complex interplay of molecular and process parameters in improving the encapsulation efficiency.

Transmission electron microscopy (TEM) was applied to investigate the morphology of the structures formed during the ionic gelation process (samples S1–S5). The analysis revealed distinct differences in structural organization depending on the chitosan–TPP ratio. As illustrated in Figure 2B,C, spherical nanocapsules with a diameter of 50 nm were solely observed in sample S5. In contrast, film-like structures were identified for the remaining samples. It should also be pointed out that sample 5 exhibited the highest encapsulation efficiency of the drug. This finding highlights once again the important influence of stoichiometric ratios and nanoscale organization on the encapsulation process. Consequently, sample 5 was selected for further studies to evaluate the anti-cancer activity of encapsulated ciprofloxacin in chitosan–TPP nanoparticles, utilizing the 3D spheroid cancer model.

**Figure 3:**
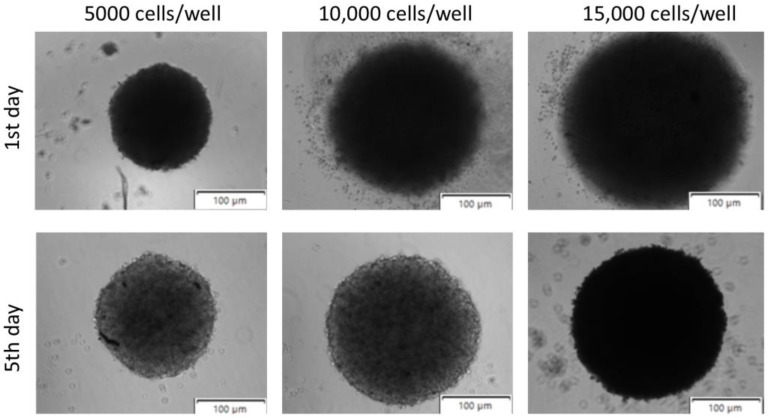
Multicellular spheroids T24 cells after 1st and 5th day after bioprinting process. All white scale bars represent 100 μm.

It is well known that the 3D model is a promising platform for novel anti-cancer drug development and screening [38]. Bladder cancer research has traditionally relied on 2D cell culture systems, with the T24 cell line being particularly valuable for studying high-grade transitional cell carcinoma. In 2D models, T24 cells have provided insights into basic cancer mechanisms and initial drug screening; however, these models lack the architectural complexity of solid tumours [39]. The transition to T24 spheroids represents a significant advancement as these 3D cultures better recapitulate the tumour microenvironment with its oxygen gradients and cell–matrix interactions [40]. Despite the substantial benefits of the 3D model over the 2D alternative, there are still many challenges to adopting this model in preclinical studies. The most significant challenges involve uniformity and reproducibility, cell growth and treatment efficiency assessment, and well-established screening procedures [38]. The type of cells, density, specific culture methods, and incubation time affect the seeding and spheroid formation procedure [40,41]. Factors such as matrix composition, mechanical properties, and oxygen/nutrients gradients also play crucial roles in spheroid development and behaviour. Although 3D models have clear advantages, they lack systemic factors such as immune response, metabolism, and organ–organ interactions, which in vivo models can provide. These limitations can be partially addressed by developing more complex systems, such as co-culture models incorporating immune cells or using perfusion systems to better mimic physiological conditions. However, for localized studies focusing on cell behaviour and drug effects within the tumour microenvironment, 3D in vitro systems are an excellent alternative or complement to in vivo experiments, particularly for understanding drug penetration, resistance mechanisms, and cell–cell interactions in a more physiologically relevant context. The present study optimized the number of spheroid-forming cells, their cell survival rate, and the time needed for spheroid growth to formulate a 3D spheroid model that mimics the bladder cancer environment. The model aimed to replicate the in vivo tumour microenvironment closely [42]. This optimization is crucial because it directly influences the model’s ability to accurately represent tumour characteristics such as cell–cell interactions, oxygen gradients, and drug penetration patterns. Figure 3 demonstrates spheroids seeded at 5000, 10,000, and 15,000 of bladder cancer cells, 1 and 5 days after procedure initiation. The results showed that the regular shape of the spheroids can be obtained when the cellular density is around 5000 cells/well. It should also be highlighted that bladder cancer cells are highly predisposed to form spheroids due to their inherent cell–cell and cell–matrix interactions, consistent with other published data [43]. This natural predisposition makes them particularly suitable for 3D culture studies as they readily form cohesive structures that better represent the tumour architecture. The use of spheroids in bladder cancer research enables a better understanding of tumour dynamics and the testing of new therapies in a setting that more closely resembles clinical reality. Additionally, these 3D models provide insights into drug resistance mechanisms and cellular heterogeneity that cannot be observed in traditional 2D cultures.

**Figure 4:**
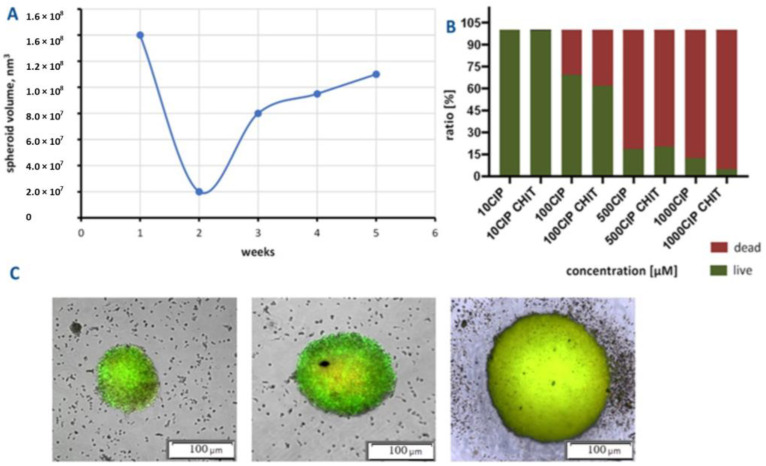
Growth dynamics and cells’ activity assessment of T24 bladder cancer spheroids used as 3D tumour models for drug efficacy evaluation. (**A**) T24 cell spheroids growth kinetics; (**B**) live/dead T24 cells treated with ciprofloxacin and encapsulated ciprofloxacin; (**C**) fluorescence imaging of T24 spheroids stained with Calcein AM. All white scale bars represent 100 μm.

The growth kinetics of the spheroids was examined at regular time intervals. Spheroids generated by magnetic bioprinting had similar growth trends measured by spheroid diameter, even when seeded at different densities. However, the spheroids generated from 5000 cells were smaller than those formed with higher cell densities (10,000 and 15,000 cells) (Figure 3). This size difference reflects the impact of the initial number of cells on the spheroid formation dynamics and the final architecture. The T24 spheroids’ size increased with time in culture as the cells proliferated and grew into a compact cellular construct. Strong cell–cell and cell–matrix interactions allowed the formation of a 3D structure of cells with a smooth surface over 5 days (Figure 4A). These interactions are critical for maintaining spheroid integrity and creating a physiologically relevant tumour microenvironment. The observed kinetics are consistent with evidence that spheroid formation and growth depend highly on the initial cell density. Higher seeding densities facilitate the establishment of intercellular contacts and extracellular matrix deposition, enhancing the structural stability of the spheroids. This correlation between initial cell density and spheroid size has been reported for various cancer cell types, including glioma and breast cancer cells [38]. Understanding these density-dependent effects is crucial for optimizing experimental conditions and ensuring reproducibility across different cancer models. Optimizing cell seeding densities is crucial for balancing spheroid size and uniformity, which are essential parameters to achieve reproducible models that closely mimic the tumour microenvironment. Uniform spheroid size is particularly important for experiments requiring long-term monitoring or when faced with specific assay limitations, such as fluorescence staining dye penetration or imaging capacity [44]. Additionally, the mechanical properties of the cellular microenvironment, influenced by extracellular matrix density, play a significant role in tumour spheroid growth. Understanding these factors is vital for developing accurate in vitro models replicating in vivo conditions [45].

To further investigate spheroid dynamics the study was extended over several weeks to investigate the growth kinetics and determine the limits of spheroid growth. The growth curve is demonstrated in Figure 4A. We observed an initial decrease in spheroid volume during the first two weeks of culture. This initial volume reduction may be attributed to cellular reorganization and compaction of the spheroid structure during the early phase of formation, rather than actual cell loss or reduced proliferation. Following this initial compaction phase, the spheroids entered a growth phase with a sigmoidal pattern between weeks two and five, consistent with the establishment of stable spheroid dynamics. The magnetic bioprinting method enables the production of T24 spheroids with a volume of 1.1 × 10^8^ nm^3^ by day 35. This substantial volume achievement demonstrates the method’s capability to generate large, stable spheroids suitable for long-term studies. The sigmoidal growth observed between two and five weeks suggests that the availability of nutrients and the removal of waste products may act as limiting factors in cell cultures (Figure 4A). These gradients, inherent to 3D cultures, mirror the physiological conditions of avascular tumour regions, where limited oxygen and nutrient supply restrict cellular proliferation, resulting in a necrotic core surrounded by a viable proliferative outer layer [46]. This stratified structure closely resembles the architecture of solid tumours, making these spheroids valuable models for drug penetration and efficacy studies. The proliferation of T24 cells that form the spheroids was visualized by fluorescence microscopy (Figure 4C). Live tumour cells were labelled with Calcein AM fluorescent dye, which can pass through the cell membrane and reveal green fluorescence when bound to intracellular free calcium ions. In contrast, dead and apoptotic cells with compromised membranes cannot retain Calcein AM. This differential staining pattern clearly visualizes cell proliferation within the spheroid structure. As illustrated in Figure 4C, T24 spheroids displayed a bright green fluorescence signal, indicating optimal metabolic activity of bladder cancer cells.

Based on the results, it can be concluded that cells inside the spheroids mimic those in the solid tumour environment and also their physical interactions [47]. This similarity extends to both structural organization and cellular behaviour patterns, making spheroids valuable models for drug testing. The obtained 3D spheroid model was conducted to determine and compare the anti-cancer potential of encapsulated and non-encapsulated ciprofloxacin. For a precise comparative analysis, only spheroids of similar size were selected for investigation. The control spheroid had a diameter size of 160.55 μm, while the control spheroid with the encapsulated drug had a size of 160.57 μm, ensuring uniformity under experimental conditions. This careful size standardization is crucial for obtaining reliable and reproducible results.

The anti-cancer activity of the encapsulated and un-encapsulated CIP was determined based on optical imaging and colorimetric labelling techniques. These complementary methods provide comprehensive insight into both morphological changes and cell activity. The capsules containing 10, 100, 500, and 1000 µM of ciprofloxacin and the pristine CIP at the same concentration were used for further studies. The concentrations of the encapsulated and non-encapsulated drugs in the experiment were the same to ensure comparability. The MTT assay evaluated the metabolic activity of spheroid cultures of the T24 cell line. The cells were stained with Calcein AM (green channel, indicating live cells) and propidium iodide (red channel, indicating dead cells). While reduction in MTT conversion is commonly used as an indicator of cytotoxicity, we acknowledge that the ultimate goal of cancer therapy is complete tumour eradication. Therefore, we complemented our MTT data with live/dead staining to directly visualize cell death.

**Figure 5:**
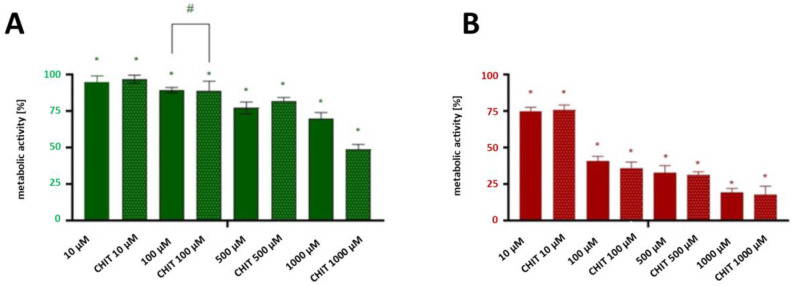
Comparative analysis of anti-proliferative activity between non-encapsulated and chitosan-encapsulated ciprofloxacin on 3D bladder cancer spheroids (**A**) Metabolic activity inhibition in 3D spheroids exposed to non-encapsulated and encapsulated ciprofloxacin in the concentration range of 10–1000 μM for 24 h; (**B**) and 72 h. Statistical significance was determined using a two-way ANOVA followed by Sidak’s post hoc test for between-group comparisons and a one-way ANOVA with Tukey’s post hoc test for concentration-dependent effects. “*”—statistical significance in relation to the control; “#”—statistically significant difference between the corresponding concentrations of ciprofloxacin and modified ciprofloxacin (*p* < 0.05).

Both forms of ciprofloxacin caused a significant decrease in cancer cells’ proliferation at concentrations higher than 500 µM (Figure 4B and Figure 5A). The encapsulated form of CIP at a concentration of 1000 µM exhibited the highest anti-cancer activity, reducing spheroid metabolic function by approximately 21% more than the non-encapsulated CIP, which suggests that encapsulation significantly improves the drug’s therapeutic potential. After 24 h of incubation, the differences between encapsulated and non-encapsulated CIP activity at a concentration of 500 µM were insignificant. However, after 72 h of exposure to encapsulated/non-encapsulated CIP, statistically significant differences between the CIP in the pristine and encapsulated form were observed (Figure 5B). At a concentration of 500 μM of encapsulated CIP the number of dead cancer cells was higher by nearly 15%. While our in vitro results show efficacy at higher concentrations and longer exposure times than might be immediately achievable in systemic circulation, the enhanced drug retention and penetration demonstrated by our encapsulation system could potentially improve efficacy in localized therapies such as intravesical administration, where direct instillation into the bladder cavity allows for higher local drug concentrations. It could also be concluded that encapsulation using materials such as chitosan provides a controlled and gradual release of the active compound and enhances cellular uptake due to its mucoadhesive and positively charged nature, which promotes interaction with negatively charged cell membranes [48]. These properties make chitosan an ideal carrier for improving drug delivery efficiency and cellular targeting. This prolonged interaction time increases the likelihood of drug penetration into the spheroid’s dense cellular structure, thereby amplifying its cytotoxic effects [49]. This increased internalization results in higher intracellular drug concentrations, potentiating the therapeutic impact on cancer cells. The enhanced drug retention and distribution within spheroids demonstrates the advantages of encapsulation in overcoming traditional drug delivery barriers. The increased cytotoxicity observed with encapsulated CIP may also stem from its ability to penetrate the multicellular spheroids better. With their 3D architecture, spheroids present a significant barrier to drug diffusion, mimicking the challenges encountered in the avascular regions of solid tumours. This barrier effect is particularly relevant for evaluating drug delivery systems intended for solid tumour treatment. Non-encapsulated CIP, with its faster release and potential rapid clearance, may struggle to achieve sufficient intracellular concentrations within deeper layers of the spheroid, limiting its efficacy over time. Encapsulation thus mitigates these limitations by maintaining higher localized concentrations of CIP, particularly in the spheroid core. This improved drug distribution pattern suggests potential benefits for clinical applications, particularly in the treatment of solid tumours with poor vascularization.

Both forms of ciprofloxacin caused a significant decrease in cancer cells’ metabolic activity at concentrations higher than 500 µM (Figure 5A), as determined by the MTT assay performed on both 2D cultured cells and 3D spheroids as described in Section 3.3 and Section 3.4.

Based on the results of MTT test, the concentration at which a CIP exerts half of its maximal inhibitory effect (IC50) against T24 cancer cells was determined. The IC50 of pristine ciprofloxacin was found to be approximately 130 μM, and for encapsulated ciprofloxacin it was estimated to be approximately 120 μM. The difference in IC50 values (representing an approximately 11% improvement) suggests a modest enhancement in cytotoxic potential through encapsulation. While statistical significance was not achieved at lower concentrations, the consistent trend toward improved efficacy with the encapsulated form, particularly evident at higher concentrations and longer exposure times, supports the potential value of this delivery system. These improvements, though moderate, may be attributed to chitosan’s unique properties as a drug carrier, including its ability to enhance drug solubility and cellular penetration [48,50]. It is important to acknowledge that the significant differences between formulations were observed mainly at 1000 μM—a concentration higher than might be physiologically relevant in standard systemic administration—which suggests that further optimization of the delivery system may be needed before clinical application. Nevertheless, these findings provide valuable proof-of-concept evidence for the potential benefits of chitosan encapsulation. While our in vitro results show efficacy at higher concentrations and longer exposure times than might be immediately achievable in systemic circulation, these parameters could potentially be relevant for localized therapies such as intravesical administration, where direct instillation of therapeutic agents into the bladder cavity is standard practice. 

**Figure 6:**
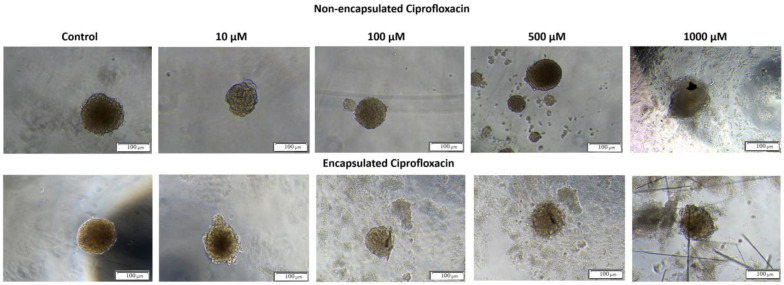
The comparison of both forms of ciprofloxacin (encapsulated and non-encapsulated) influencing cell growth of the 3D bladder cancer cell spheroid model. All white scale bars represent 100 μm.

The optical microscopic observation of 3D spheroids incubated with non-encapsulated and encapsulated CIP confirmed that untreated 3D spheroids exhibited spherical shape and smooth surfaces (Figure 6, control spheroids). The morphological changes were registered for 3D spheroids exposed to encapsulated and non-encapsulated CIP. However, smaller and more irregular spheroids with many individual cells at the surface and around the spheroid were visible for encapsulated CIP at concentrations higher than 10 µM (Figure 6). These results indicate that T24 spheroids are more responsive to encapsulated ciprofloxacin than non-capsulated ones, suggesting that encapsulation may improve drug distribution and cellular accessibility within the spheroid structure.

**Figure 7:**
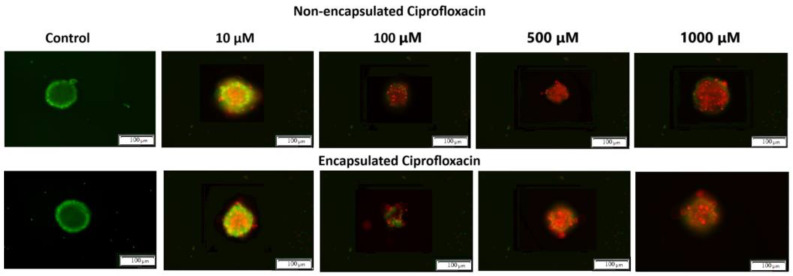
CLSM (confocal laser scanning microscopy) imaging of T24 spheroids treated with ciprofloxacin and encapsulated ciprofloxacin after 24 h of incubation. All white scale bars represent 100 μm.

Additionally, the results were analyzed using artificial intelligence tools to quantify and evaluate the morphological changes. The image analysis was conducted using the Count and Measure module of the Cellsens Dimension software version 3.2 (Olympus Cooperation, Tokyo, Japan). It consists of detecting objects using the “manual threshold” method and counting the detected objects with which cells were stained. The artificial intelligence tools used in the study included a neural classifier based on the U-NET network, implemented in the Cellsens Dimensions software package, which enabled automated image analysis and cell classification based on their morphological features. Cells marked as “live” (green field) and “dead” (red field) were detected (Figure 7).

The untreated 3D spheroids (control) revealed green fluorescence, indicating intact structural integrity with viable cells. Upon exposure to both formulations of ciprofloxacin, concentration-dependent morphological alterations became evident. At lower concentrations (10 μM) both formulations showed a mix of live (green) and dead (red) cells, while at higher concentrations (500–1000 μM) dead cells predominated, as indicated by intense red fluorescence. Notably, the shape of spheroids treated with encapsulated CIP appeared more irregular compared to those treated with non-encapsulated CIP, particularly at higher concentrations. This enhanced morphological disruption suggests deeper penetration and more extensive distribution of the encapsulated drug throughout the spheroid structure. At the highest concentration (1000 μM), both formulations resulted in extensive cell death, with viable cells being almost undetectable. These findings demonstrate that both formulations exert effective anti-cancer activity, with encapsulated CIP causing more pronounced structural disruption, suggesting it better overcomes the diffusion barriers typically present in solid tumours.

## 3. Materials and Methods

### 3.1. Materials

Chitosan (MW = 190,000–310,000 Da, 1% in 1% acetic acid (ethanoic acid) aqueous solution), sodium tripolyphosphate (pentasodium triphosphate, TPP, 85 %), ciprofloxacin (>98%), phosphate-buffered saline (PBS, 0.01 M, pH 7.4), and thiazolyl blue tetrazolium bromide (3-(4,5-dimethylthiazol-2-yl)-2,5-diphenyl tetrazolium bromid, MTT) were purchased from Sigma Aldrich (Sigma-Aldrich Chemie GmbH, Steinheim, Germany). Sodium hydroxide (ACS reagent, ≥98.5%, pellets, for synthesis) and trypan blue solution were purchased from Scharlau (Sentmenat, Spain). Hydrochloric acid (37%) and a Fluorescence-based LIVE/DEAD™ Cell Imaging Kit were purchased from Thermo Fisher Scientific (Waltham, MA, USA), while magnetic nanoparticles NanoShuttle™ were obtained from Greiner Bio-One (Kremsmünster, Austria)). Trypsin solution, ethylenediamine tetraacetic acid (*N*,*N*′-(Ethane-1,2-diyl)bis[*N*-(carboxymethyl)glycine], EDTA), and trypan blue were purchased from Corning (Corning, NY, USA). Dimethyl sulfoxide (DMSO) was purchased from StanLab (Lublin, Poland). All the chemical compounds were used as provided without any further purification.

### 3.2. Preparation and Characterization of Chitosan Nanoparticles

The chitosan nanoparticles were produced via the ionic gelation technique [51]. Firstly, chitosan (0.1–1%) was dissolved in 3% *v*/*v* acetic acid, and the pH of the solution was adjusted to 4.7 using 1 M NaOH. Then, TPP solution (0.1–0.5% *w*/*v*) was prepared. After that, ciprofloxacin (1 mM) was dissolved in a PBS solution, in which the pH was also adjusted to 4.7. Then, a mixture of the drug and TPP solutions (1:9 *v*/*v* ciprofloxacin solution: TPP solution) was added dropwise to the chitosan solution (the volume of chitosan solution employed was three times higher than the volume of the drug–TPP mixture). The final suspension was stirred at 350 rpm for 30 min. The solution was centrifuged at 13,000× *g* at 4 °C for 30 min to collect the nanoparticles. The purified nanoparticles were dried or suspended in an aqueous solution.

Chitosan used for nanoparticle preparation was characterized by ^1^H NMR spectrum, which was recorded in a 2 wt.% solution of deuterated acetic acid (CD_3_OOD) in deuterated water (D_2_O) with a Bruker Avance Neo 400 MHz spectrometer (Bruker BioSpin GmbH, Rheinstetten, Germany) (^1^H—400 MHz) at room temperature (25 ± 5 °C). The central peak of D_2_O was taken as the reference (4.79 ppm). The spectrum was registered using an acquisition time of 4 s, and the number of transients was equal to 32 scans with a pulse delay time of 1 s.

The size and morphology of the nanoparticles were determined using transmission electron microscopy (TEM) JEOL JEM1011 (JEOL USA, Inc, Peabody, MA, USA).

The encapsulation efficiency of the drug in nanoparticles was estimated by UV spectrophotometry (Spectrophotometer UV-1800, Shimadzu Corporation, Kyoto, Japan) (λmax (ciprofloxacin) = 277 nm). The amount of encapsulated drug was calculated using the following Equation (2):

(2) $$EE\left(\%\right)=\frac{CIP_{0}-CIP_{t}}{CIP_{0}}\cdot 100$$

where CIP_0_ (mg/L) represents the initial concentration of the drug and CIP_t_ (mg/L) represents the concentration of the drug in the supernatant.

### 3.3. Cell Culture Assessments

All procedures regarding cell culture were carried out using a class II laminar flow cabinet (Bio II Advance—Telstar Technologies, Terrassa, Spain). Cell passage was performed under sterile conditions in a type II laminar chamber. It was carried out after the cells achieved approximately 60–80% of culturing area coverage. In the first step, the medium was removed and the cell growth surface was washed with PBS without Ca^2+^ and Mg^2+^ ions. In the next step, 1.5 mL of a 0.05% trypsin solution with EDTA was added per 25 cm^2^ culture bottle to the T24 cells. The cell detachment process, performed for 2–5 min at 37 °C, was monitored under a microscope with reversed optics (Nikon Corporation, Tokyo, Japan). An equal volume of the culture medium was added to the culture flask to inactivate trypsin. The cell suspension was transferred to a sterile tube and centrifuged for 5 min at 2500 rpm. After centrifugation, the supernatant was decanted and the cell pellet was resuspended in an appropriate culture medium. The number of viable cells was calculated using a Neubauer chamber using trypan blue. Then, 25 µL of the cell suspension was added to 25 µL of 0.4% filtered trypan blue solution. The cell suspension and trypan blue solution were applied to the Neubauer chamber. Cells were counted using an inverted optics microscope. Viable cells were counted from the 4 squares of the chamber, and their total number was calculated by Equation (3), which is as follows:

(3) $$L=\frac{A}{4}\cdot 2\cdot 10^{4}\cdot B$$

where L is the total number of cells, A is the number of live cells counted from four squares, and B is the volume of the medium (mL).

Morphological evaluation of cells was performed using an inverted-phase contrast microscope (CKX53-FL, Olympus Corporation, Tokyo, Japan) and a dedicated 4K colour camera (UC90, Olympus Corporation, Tokyo, Japan). Image analysis was carried out at intervals, i.e., at the beginning of experiment and after 24 and 72 h. At each of the time points the documentation of the examined cells was made. A fluorescence-based LIVE/DEAD™ Cell Imaging Kit assay was used to examine cell viability. Green fluorescent Calcein AM (ex/em 488 nm/515 nm) and red fluorescent DNA stain (ex/em 570 nm/602 nm) were used to precisely determine live and dead cells. Live cells were bright green, whereas dead cells with compromised membranes were fluoresce red. After 24 h incubation, encapsulated and non-encapsulated ciprofloxacin were added to wells, and cells were cultured for the next 72 h. Cell staining was performed following the manufacturer’s protocol. Labelled cells were observed under a fluorescence microscope (IX83, Olympus, Japan). Microscopic images were used for subsequent quantitative analysis (Cell Sens Dimension, Olympus, Japan). Image analysis was performed in the Count and Measure module dedicated to Cellsens Dimension software (Olympus Corporation, Tokyo, Japan). It consisted of detecting objects using the “manual threshold” method and counting the detected objects with which apoptotic cells were stained.

Cell metabolic activity in both 2D and 3D culture systems was assessed using the thiazolyl blue tetrazolium bromide (MTT) assay (Sigma-Aldrich Chemie GmbH, Steinheim, Germany). Following incubation periods, the culture medium was removed and replaced with 100 μL of fresh medium containing 0.5 mg/mL MTT solution. Plates were incubated for an additional 4 h at 37 °C to allow formazan crystal formation. The medium was then carefully aspirated, and the formazan crystals were dissolved in 100 μL DMSO. After 10 min of gentle shaking to ensure complete dissolution, absorbance was measured at 570 nm with a reference wavelength of 650 nm using a microplate reader (iMark™ Microplate Reader, Bio-Rad, Philadelphia, PA, USA). Cell metabolic activity was calculated as a percentage relative to untreated control cells. Each experiment was performed in triplicate.

### 3.4. Preparation and Characterization of Multicellular Tumour Spheroids

The spheroids model was made using a magnetic bioprinting method. The magnetic nanoparticles NanoShuttle™ were biocompatible and did not disturb the metabolism and cell proliferation. Briefly, the cancer cells were seeded in a 24-well plate, and when a 60% state of confluence was reached the magnetic nanoparticles were added and incubated overnight. After 24 h, the obtained cells containing magnetic nanoparticles were counted and seeded on 3D plates (NanoCulture 96-well Plate^®^ (NCP-LH-96); SCIVAX, Kanagawa, Japan) at a density of 5 000, 10,000, and 15,000 cells/well to optimize the cell number needed for spheroids formation. Then, the bioprinting process was performed. Thiazolyl blue tetrazolium bromide was used to measure cell proliferation as a function of redox potential according to ISO 10993–5:2009 [52].

For spheroids culture, mature spheroids grown from 5000 T24 cells on 3D plates (NanoCulture 96-well Plate^®^ (NCP-LH-96); SCIVAX, Kanagawa, Japan) were incubated with encapsulated and non-encapsulated ciprofloxacin for 24, 48, and 72 h, after which the medium was replaced with MTT reagent (1 mg/mL) for at least 2 h at 37 °C. Next, the culture medium was aspirated and the remaining formazan was dissolved in DMSO. The absorbance was measured at 570 nm and a reference wavelength of 650 nm using a microplate reader (iMarkTM Microplate Reader BIO-RAD, Philadelphia, PA, USA). Each experiment was performed at least in triplicate. After all replicates were performed, the results were used to calculate the lethal concentration (L.C.) causing 10, 50, and 90% cell death—LC10, LC50, and LC90. The curve equation was generated based on the obtained results, allowing for the appropriate calculation of the L.C. value.

Spheroids kinetics growths were observed, captured, and evaluated with a live imaging system for 6 weeks after initial cell seeding (day 0) using a BZ-X710^®^ inverted microscope (Keyence, Osaka, Japan). Morphometric analysis of spheroids and measuring their area was performed using EPview 1.3 software (Olympus Corporation, Tokyo, Japan). Growth kinetics were being investigated by image analysis using a pixel classifier based on a dedicated U-NET neural network.

In order to guarantee the correct operation of the classifier, identical imaging conditions were necessary when preparing the training database (Ground Truth) and the proper study of growth kinetics. A motorized one was used for the imaging inverted microscope IX83 (Olympus Corporation, Japan) adapted to this type of acquisition, equipped with a monochrome camera (Hamamatsu Orca Spark). Imaging was performed in 96-well plates with a plastic flat bottom. The hare of featured photos in network training and analysis was carried out using the high-contrast brightfield technique, which ensured there were no shading artefacts present when using the contrast technique phase with this type of culture vessel. The neural network-based classifier was trained using the Cellsens Dimensions package and AI. The training database contained over 100 photos of cells made at different stages of proliferation. In the images, pixels belonging to the following three classes of objects were manually marked with high accuracy: background, cells, and cells in the division process (having a circular morphology). Then, training a neural network based on U-NET was performed with the use of hardware acceleration (GPU Quadro p2200) and took at least 100,000 network iterations until over 97% success was achieved. The generated classifier was then validated for accuracy on a new pool of images on which it was not trained.

Statistical analysis was performed using GraphPad Prism version 8.4 (GraphPad Software, San Diego, CA, USA). Each experiment was performed at least in triplicate. The average cell survival rate was expressed as a percentage relative to the control. All data were presented as means ± S.D. The normal distribution of data was analyzed using the Shapiro–Wilk test. Parametric analysis was performed with a one-way ANOVA with Tukey post hoc (for cellular proliferation) or a two-way ANOVA with Sidak post hoc (for grouped analysis). Non-parametric analysis was made based on the *t*-test with the Mann–Whitney post-test. This study assumed that the *p*-value, i.e., the test probability, must be less than 0.05 for the difference to be considered statistically significant.

## 4. Conclusions

This study demonstrates the enhanced anti-cancer potential of chitosan-encapsulated ciprofloxacin in a 3D spheroid model of bladder cancer, offering a promising approach to overcome the limitations of traditional drug delivery systems. The encapsulation significantly improved the drug’s cytotoxic effects compared to free ciprofloxacin, highlighting the advantages of biopolymer-based nanocarriers in cancer therapy. The study shows the impact of chitosan–TPP ratios on the morphology and ciprofloxacin encapsulation. The results indicated that encapsulation efficiency increased with decreasing [${-NH}_{3}^{+}$]/[O^−^] ratio. This optimization of the encapsulation process is crucial for developing effective drug delivery systems.

The anti-cancer potential of the encapsulated drug was investigated in a 3D bladder cancer cell spheroid model, which better mimics in vivo conditions than 2D cancer models. These 3D models provide valuable insights into drug penetration and efficacy that cannot be obtained from traditional monolayer cultures. Spherical chitosan nanocapsules containing ciprofloxacin demonstrated enhanced anti-cancer activity against T24 bladder cancer cell spheroids compared to non-encapsulated drugs. Significant cytotoxic effects of encapsulated ciprofloxacin at concentrations of 500 and 1000 μM were observed in vitro after 48 and 72 h of exposure. These results indicate a time- and concentration-dependent response that may be consistent with a sustained release profile. However, maintaining constant drug concentrations in vivo over such extended periods is challenging and requires further investigation. Additional studies, including clonogenic assays and in vivo pharmacokinetic analyses, are necessary to confirm the therapeutic potential and clinical applicability of encapsulated ciprofloxacin in bladder cancer treatment. 

The results indicate that nano-encapsulated ciprofloxacin exhibits higher cytotoxicity than the free form of the drug, suggesting that the proposed approach may be more effective in treating bladder cancer. The enhanced efficacy could potentially lead to reduced drug doses and fewer side effects in clinical settings. The study also confirms that the combination of ciprofloxacin with chitosan increases the specificity and efficacy of the therapeutic effect. However, future research regarding the long-term stability of these encapsulated formulations should be explored to realize their full potential in preclinical applications.

## Figures and Tables

**Table 1:** Composition and encapsulation efficiency of ciprofloxacin nanocapsules prepared with varying chitosan and TPP ratios.

Sample	[Chitosan] (% w)	[TPP] (% w)	Ciprofloxacin (% w)	Ratio $[{-NH}_{3}^{+}$]/[O^−^]	Ratio m_Chitosan_/m_TPP_	Encapsulation Efficiency (%)
S1	0.10	0.18	0.032	0.8	1.6	16
S2	0.40	0.50	0.032	1.2	2.4	9
S3	0.60	1.00	0.032	0.9	1.8	0
S4	0.16	0.10	0.032	2.4	4.9	9
S5	0.10	0.50	0.032	0.3	0.6	22

## Data Availability

Data will be made available on request.
